# Laying Grounds for Dialogue: Exploring Anti‐Racist Activists' Negotiations of Emotions When Challenging Colour‐Blindness in Norway

**DOI:** 10.1111/1468-4446.70121

**Published:** 2026-05-03

**Authors:** Kine Marie Michelet

**Affiliations:** ^1^ Department of Social Work Child Welfare and Social Policy Oslo Metropolitan University Oslo Norway

**Keywords:** affective economies, anti‐racist activism, coloniality, colour‐blindness, emotions, ideology

## Abstract

In this article, I explore how 36 Norwegian anti‐racist activists of colour negotiate emotions when engaging with the white majority population. Much recent research on racist ideology draws on Bonilla‐Silva's framework of colour‐blindness, arguing that the white majority nowadays is more likely to deny systemic racism. However, few studies have explored the relationship between micro‐level interactions and colour‐blind ideology beyond the realm of language. Using Ahmed's theory on affective economies along with decolonial perspectives on emotions, I empirically demonstrate how an unequal distribution of emotions across racial divides works as a central mechanism in the development of colour‐blind ideology. This finding reveals the need to incorporate the role of emotions within the literature on colour‐blindness more broadly, as similar tendencies may be prevalent in other Western societies.

## 1 Introduction

Much recent research on racist ideology draws on Bonilla‐Silva's (2021) framework of colour‐blindness, explaining how the white majority nowadays is more likely to deny systemic racism. Key to this argument is that the language of colour‐blindness is slippery, inconsistent, and often subtle, thus contrasting with the explicit racial language used during the Jim Crow era. Studies building on this understanding have mainly focused on the underlying logic and historical development of colour‐blindness, underscoring its persistent and stable nature (Burke 2016; Doane 2017; Lentin 2020; Lipsitz 2019). Yet, one perspective has been left out within this comprehensive literature: The emotional experience of people resisting such domination. Considering the embodied impact of being subjected to racism, as well as the increasing need for facilitating constructive dialogue on this issue, this neglect is remarkable.

Indeed, there is no reason to believe that language does not play an essential role in (re)producing colour‐blind ideology. Nor does Bonilla‐Silva (2019) himself reject the importance of emotions in the study of racist ideologies. Still, the literature has not adequately addressed the precise ways emotions work within the framework of colour‐blindness. By using Ahmed's (2004) theory on ‘affective economies’ alongside decolonial perspectives on emotions, I understand emotions as relational and a form of economy. These perspectives help us unpack why communication about racism across racial divides often fails, by highlighting how it is the emotional attachment itself that aligns some individuals with or against others. As emotions essentially shape ontological realities, an unequal distribution of emotions across racial divides may therefore, on their own, disrupt communication at a more fundamental level.

This article explores how anti‐racist activists of colour negotiate emotions when engaging with the white majority population. Based on the argument that we gain unique insight into systems of oppression by exploring attempts to challenge them (e.g., Collins 2000), my analysis draws on interviews and fieldwork with 36 anti‐racist activists of colour. The interviews demonstrate that both negative *and* positive emotions expressed by the white majority play an essential role in (re)producing colour‐blindness, while also reflecting colonial dynamics that delineate the boundaries between the human and the Other. This finding reveals that anti‐racists deal with subtle, confusing, yet violent social interactions that would remain unrecognised through an analytical focus on language alone. Nevertheless, they are resisting such domination by continuously negotiating whose emotions should be considered most valid, thereby laying grounds for constructive dialogue.

This article contributes to the literature on racism by exploring how emotions work as a central mechanism in the development of colour‐blind ideology in a seemingly colonial innocent society. I demonstrate that the anti‐racists navigate both colonial dynamics of emotions and contemporary racial expressions simultaneously, thereby contributing to a deeper understanding of the relatively persistent and stable nature of colour‐blindness (e.g., Bonilla‐Silva 2021). Furthermore, as Norway has never recognised its colonial past as part of the national narrative like most other Western countries, this study highlights the potential of challenging the economy of emotions as a sustainable form of resistance that remains resilient to the constant and ever‐shifting flexibility of the language. On this matter, it is important to recognise that tendency of claiming colonial innocence is prevalent across all Western societies. Hence, these findings call attention to include emotions within the framework of colour‐blindness more broadly, potentially offering fresh insights into possibilities of change.

I start out by situating this study within the broader framework of colour‐blindness, as well as discussing Ahmed's theory of affective economies and related theories on emotions. Before presenting the data‐material and methodological reflections related to conducting qualitative interviews with anti‐racist activists, I describe the national context in which participants' stories unfold. Based on the analysis presented in three parts, I conclude by highlighting the need to acknowledge emotions to facilitate constructive dialogue on racism.

## 2 Theoretical Perspectives

### 2.1 Colour‐Blind Racism, (Counter) Narratives, and the Importance of Context

In the literature, the concept of colour‐blindness has gained significant recognition as the dominating way of expressing racist ideology in contemporary Western societies (Burke 2016; Doane 2017; Lentin 2020; Lipsitz 2019). Much of this research builds on Bonilla‐Silva's framework (Bonilla‐Silva 2015, 2021), explaining how racist ideology is often expressed though the denial of structural racism. Key to his argument is that nowadays, few whites would claim to be racist. Nevertheless, racial inequality persists, although often framed as subtle, institutional, and apparently non‐racial. Several scholars have detailed the persistent and stable nature of colour‐blind ideology; yet, important gaps remain in the understanding of the specific mechanisms and internal dynamics embedded within the concept itself (Doane 2017; Hughey et al. 2015).

Bonilla‐Silva (2021, 77) argues that: ‘subscribing to an ideology is like wearing a piece of clothing. When you wear it, you also wear a certain style, a certain fashion, a certain way of presenting yourself to the world’. This understanding reminds us that our way of thinking is not only shaped by the ideology surrounding us, but through narratives and storylines, we are also actively shaping ideologies. A key aspect of this argument is that the linguistic manners, rhetorical strategies and technical tools, embedded within the narrative processes itself, (re)produce colour‐blind ideology. Nowadays, the language of expressing racism is flexible, often subtle, and apparently contradictory, thus contrasting to the discourse during the Jim Crow era. While previous research has examined the underlying logic and historical development of colour‐blindness—with many arguing that whites are in a better position to deny systemic racism today (e.g., Alexander 2012), few studies have paid attention to dynamics of change, and even fewer how the relationship between micro‐level interactions and racist ideology may extend beyond the realm of language.

This raises the question of what it means to tell an alternative story that challenges the dominant narrative. Race scholars have long addressed how counter‐narratives offer unique insights into systems of oppression (e.g., Collins 2000; hooks 1982); however, research exploring the narrativisation of resistance against colour‐blind ideology is surprisingly scarce (van Dijk 2021). Evans and Moore's (2015) study on resistance among people of colour within institutional spaces is, however, an insightful exception. A takeaway here is that participants, in navigating racial and colour‐blind discourses at their workplace while simultaneously attempting to achieve success, are experiencing an unequal distribution of ‘emotional labour’. While this concept originates from feminist scholarship (Hochschild 2012), showing how women suppress feelings to please the patriarchy, race scholars have shown how similar tendencies extend to racial dynamics (e.g., Emejulu and Bassel 2024; Evans 2013; Mirchandani 2003; Wingfield 2010, 2021), where racialised ‘feeling rules’ that dictate what and how one should feel constitute domination through the repression of emotional expressions.

While research emphasises the need to move closer toward a global theory of colour‐blindness (Beaman and Petts 2020; Doane 2017)—noting that ideologies always are part of a broader constellation of ideas beyond national contexts—most empirical studies are still situated in US and UK. Yet, there are no reasons to believe that colour‐blind ideology is exclusive to these contexts (Garner 2014; Lentin 2020). For example, by bringing together existing research in the US and France, Beaman and Petts (2020) argue that colour‐blindness in France is not just about denying structural racism; it also means rejecting racial and ethnic categories. Building upon these scholarly insights, this current study demonstrates the importance of incorporating emotions within the literature on colour‐blindness more broadly.

### 2.2 ‘Affective Economy’, Entitlement to Enjoyment, and Racialised Emotions

While narratives resisting colour‐blindness help us unpack how whites discursively incorporate evidence from their everyday life to deny systemic racism, emotions help us understand why such narratives are activated in the first place (Meghji 2022). Over the past decades, the role of emotions has received increasing attention from activist and social movement scholars exploring dynamics of change (e.g., Emejulu 2025; Hemmings 2012; Liu and Shange 2018). This ‘affective turn’ is largely driven from critical race theory, queer, and feminist scholarship (e.g., Butler 2024; Fanon 2008; Hochschild 2012), highlighting that emotions are not just an individual matter, but work as a constitutive force in shaping ontological realities, politics, and society. In joining this tradition, this study demonstrates how different emotions across the racial line cement the divide in understanding found on a discursive level.

Ahmed's (2004) theory on affective economies provides three distinct and important insights for this analysis. The first one is that emotions work as an economy, as they circulate accumulate, and attach to certain bodies and spaces. In addition to highlighting how emotions *do* things, by working in concrete ways to include some and exclude others, this argument underscores how emotions essentially are relational. Emotions do not simply exist ‘within’ or ‘without’; rather, it is the intensity of the emotional attachment itself that align individuals with or against others and the world. However, emotions are not equally distributed. As emotions tend to ‘stick’ more to some bodies, different sets of emotional constellations across the racial line may therefore, on their own, trouble communication and mutual exchange.

Decolonial scholarship deepens our understanding of how the unequal distribution of emotions ultimately stems from the colonial dichotomy between the human and the Other, highlighting that the category of human essentially is a construction of whiteness (e.g., Fanon 2008; Moten 2003; Sharpe 2016; Yancy 2008). One scholar who illustrates this well is Hartman's (2024) analysis of the interconnection of the enslavement and present‐day racism. A key element of what she terms ‘scenes of subjection’—the routine practices through which Black suffering was spectacularly and subtly displayed, is the notion of entitlement to enjoyment. She details how Black people were forced by slaveowners to engage in performative acts of joy, such as singing and dancing, to make racial oppression appear less brutal. This emphasises how violence not only involves physical punishment but is equally about control of emotions, subjectivity, and stripping away humanity. Similarly, this current study argues that similar colonial dynamics play out in a colour‐blind context like Norway, though shifting depending on the context and appearing more innocent.

Putting Hartman in conversation with race scholars of emotions therefore offers opportunities to remain attentive to specific Western national contexts *and* its underlying colonial and global linkages (Meghji 2023). Similar associations may be what Bonilla‐Silva (2019) alludes to in his later work when noting he has been ‘feeling race’ his entire life. Adding depth to his structural approach, he proposes a theoretical sketch on racialised emotions which he describes as the ‘socially engendered emotions in racialized societies’ (2019, p. 3). From this perspective, race, emotions and ideology are intertwined, precisely because race is related to and shapes emotions that generally matches the location in the racial order. Combined, these contributions call attention to the need to retheorise how colour‐blindness operates and manifests through various emotional states, as well as being negotiated and contested through everyday practices.

## 3 Context: ‘Nordic Exceptionalism’ and the Search for Language

This study was conducted in Norway, a country which is often perceived—both nationally and internationally—to be innocent of historical and contemporary colonial practices (McEachrane 2014). While the myth of ‘Nordic exceptionalism’ has been debunked from various angles, Norway's national identity still heavily relies on narratives about egalitarianism, gender equality, and its role as a global leader in peace negotiations. Within this historical context—where a reckoning of the colonial past is absent—talking about race is highly contested, and even the existence of racism has been questioned both in academia and the public sphere until very recently (Andersson 2018).

In line with scholars highlighting the silencing of race in Europe (Lentin 2020; Song 2018), recent scholarship in Norway (e.g., Führer 2021) has raised questions of how we can develop a language that addresses differences in a context where race is not recognised as a marker of difference. Despite these concerns, an exploration of how Norwegian anti‐racist activists of colour—the group most experienced in navigating and contesting racial language—navigate this challenge themselves has not yet been scrutinised. These insights are valuable, especially considering how activists and social movements often are seen as a catalyst for exploring dynamics of change (e.g., Bassel and Emejulu 2017; Taylor 2016).

## 4 Methodological Choices and Reflections

The data material of this project was obtained in 2021 and 2024, consisting of 36 in‐depth interviews (about 90 min) with anti‐racist activists of colour along with 6 months of fieldwork where I attended workshops, cultural events, public talks, and protests in Oslo, Norway. This article specifically examines how the anti‐racists negotiate emotions when engaging with the white majority based on the interview data, however, observations from participants' activist communities and milieus have been crucial in shaping the interpretations.

Participants had diverse racialised experiences, identifying as Black/African, Asian, Latin‐American, or as from the MENA‐region or the Balkans. While almost all engaged in overlapping anti‐racist work, about one third were mainly affiliated with a public institution or employed in a state‐founded organisation, one third were freelancers and artists, and the rest worked independently with either community facilitation, grassroot activism, or rights‐based work. Their age spanned from 25–70 years, and about half of them identified as women.

Because anti‐racists activists are considered a difficult‐to‐reach population (e.g., Bassel and Emejulu 2017), I initiated the recruitment process by contacting people I had already built trust with from previous work with different anti‐racist communities. This strategy was further combined with snowball sampling by which I contacted people directly, based on connections acquired through fieldwork and recommendations from interviewees. Later on, I contacted actors who have been highly visible in the public sphere over time—such as through pioneering civil‐rights advocacy, influential artistic work, and media appearances.

The concept ‘epistemic exploitation’ describes how marginalised people often are compelled to educate privileged people about the nature of their own oppression (Berenstain 2016). Because academia represents a white institution (Ahmed 2012), I was therefore not surprised that several participants expressed a certain ambivalence when I, in the role of a researcher, asked them to share their reflections about racism with me. This partly placed me as an outsider during the recruitment phase, despite my racialised insider status and familiarity with anti‐racist work. Still, most participants agreed to take part, and throughout our conversation, they quickly put their guard down. This illustrates how the researcher is not either an insider nor an outsider, but must constantly navigate the complexity of being perceived as both (Carling et al. 2014).

While I prepared a semi‐structured interview‐guide aiming to explore experiences of talking about racism in public spaces, online interactions, within anti‐racist communities, and in their private lives, I quickly observed that utilising my own feelings as an instrument, allowed me to create a collaborative space within the research setting (Hemmings 2012). For example, a recurring topic during the interviews was the value of ‘venting out’ frustration with other people of colour, along with the joy that comes from gaining new insights. Likewise, as an Asian women growing up in Norway, I often felt interest and excitement for learning while listening to them—feelings that created drive, new directions, and a shared sense of meaning. Though, being young and less experienced with anti‐racist work, put me in a position where I could ask for explanations and elaborations, without it creating a distance.

Considering the validity and reliability of the data, it is worth noting that participants may perceive involvement in this study as a way of engaging in anti‐racist work itself. This could lead them to respond in ways that aligned with the overall message they wished to convey to a wider public, rather than their ‘true’ feelings. Some might express more clear‐cut arguments, aiming to convince an imagined white audience, while others might downplay their own anger and frustrations. However, these situations were easily recognisable to me, as the emotional intensity between us swiftly faded and made the conversation rather disengaging. To counter these situations, I responded in ways that either revealed my own true emotions or sought to evoke theirs on the topic we discussed. Most participants accepted this invitation, which provided access to a group that is otherwise challenging for researchers to get close to.

Although my analytical focus initially was to explore the relationship between narratives and colour‐blind ideology, an understudied field in Norway, I noticed that emotions were equally important while transcribing and coding the interviews. Hence, the analytical process developed inductively as I started out by grouping various emotional qualities, such as ‘sadness’, ‘anger’, ‘shame’, ‘joy’, ‘pride’, ‘excitement’ into categories. These allowed for an observation of the context in which they arose, thus resulting in three main stories of how emotions, racial power, and counter‐narratives are intertwined. Following the wishes of most participants, I further ensured their anonymity by assigning them pseudonyms. While striving to preserve as many identity markers as possible, it was, however, also necessary in some cases to change participants migration background, gender, and engagement in anti‐racist work.

## 5 ‘We Don't Agree on the Foundation’: Mapping out the Emotional Terrain of Colour‐Blindness

When challenging colour‐blind discourses in Norway, the anti‐racists observed that regardless of how carefully they chose their words, pedagogical tools they employed, or thoroughly explained the systemic and harmful impacts of racism, conversations often failed to progress in ways they perceived as constructive for change. As such, participants were less concerned with navigating language when engaging with the white majority. Nor did they really seem to care too much about the precise meaning of the words itself, as the interviews revealed they often would adjust their choice of words depending on the context. Mona's relationship with her friends of colour illustrates why: ‘Even if I use the wrong word or imprecise language, I know that [my friends] understand what I mean. We see the same monster—agree that it exists and causes harm. Then, suddenly, it becomes much easier to talk’. Likewise, when I asked Sophia whether she consider herself to have adequate vocabulary for talking about racism, she rejects my premise by saying:

Generally speaking, words come very naturally to me. I feel like I have a connection with my brain and don’t need to think much before I formulate my thoughts. But when I talk about racism, it requires something different from me. I pause more, think more, stumble over my words more than I’m used to. And then I wonder, why is that? Is it the inadequacy of the Norwegian language, or the lack of a fundamental understanding of racist structures? We don’t agree on the foundation, and that makes it difficult to have conversations.

By taking seriously Ahmed (2004),  (2014) argument that emotions constitute ontological realities, perspectives like these—where a ‘lack of a fundamental understanding of racist structures’ is framed as the primary barrier to communication—suggest that narratives supporting colour‐blind ideology are activated through something else more fundamental than language alone. Like Yosuf further illustrates, when describing why it is painful to talk about his experiences with racism as a Muslim in Norway—a country that, like other European countries (see Beaman and Petts 2020), rejects racial and ethnic categories: I feel that, in Norway, it's almost not okay to say the sentence ‘I, as a Muslim man’, because you're not supposed to acknowledge that Muslim men exist. […] And precisely because we fail to recognise the reality that is right in front of us, we also lack the language to talk about it’.

Such understandings seem to bolster how participants appear more concerned with managing and navigating their own and others' emotions when challenging colour‐blind discourses. Ada, a Black woman, illustrates this quite well. She has spent years raising awareness about the unintended and institutional forms of racism in the education sector, and reflects on how the emotional work of constantly navigating accusations of ‘reversed racism’ has become increasingly depleting: ‘When I talk to white people, I tone it down. I focus on speaking in a very warm and inviting manner so that I'm not seen as aggressive. I'm very aware of how I speak. I don't get emotionally activated’. The emotional labour preformed in interactions like these, speak to how participants sometimes adhere to racialised ‘feeling rules’ in attempts to challenge colour‐blind ideology within institutional spaces (see Evans and Moore 2015), while those very efforts reproduce racial power dynamics that made such labour necessary in the first place (e.g., Hochschild 2012; Wingfield 2021). However, navigating the emotional terrain of colour‐blindness emerged as multilayered and complex, extending beyond interactions in which the white majority actively dismisses participants concerns. As Tai, employed in a state‐founded organisation remarks when sharing personal stories of racism:

If you’ve held a lecture about racism for white people, they’re very quiet and attentive—they don’t say anything. They might not even dare to ask questions. After you’ve spoken, they’re so drained they don’t know what to do with themselves. I feel like this is a problem. But if you give the same lecture to an audience with racialised minority background, suddenly there’s so much they want to say afterward. They would share their experiences, saying, ‘I could really relate to that,’ or ‘that was different from what I’ve experienced’.

Interestingly, it is the silence following the lecture that is perceived as the main challenge in Tai's narrative, rather than sharing his own experiences of racial abuse itself. This illustrates an additional aspect of the reproduction of colour‐blindness, showing how emotions work as an economy by circulating differently between bodies (Ahmed 2004). Like Tai describes, sharing personal stories creates an emotional attachment between him and the racialised minority group, enabling meaningful conversations that challenge individualised understandings of racism. When conversations fail to proceed as intended when lecturing for the white audience, by contrast, this illustrates how lack of circulation of emotions by its own work to exclude.

Emily recognises the same pattern, although being more explicit about why the anxiety, insecurity, and discomfort she often encounters frustrate her: ‘This is my trauma we're talking about. And for white people, it's the anxiety of saying something wrong (laughs). But we're used to white pain being ranked so much higher than all other pain in the world, right?’. Statements like these not only speak to how emotions function as an exclusionary force when the anti‐racists initiate conversations on racism. It also demonstrates how the unequal distribution of emotions across racial divides unfolds within a colour‐blind context in which the majority claim liberal values (Ahmed 2004). In this economy, the fear of potentially being perceived as racist outweighs the suffering and pain of those subjected to racism, while remaining invisible to the dominant other. The centring of whites' emotions in interactions like these thus reveal how the unequal distribution of emotions disrupt communication on a more fundamental level, essentially preventing conversations from progressing in meaningful ways that may facilitate change.

## 6 ‘Fun Chats About Racism’: Navigating the Racial Power of Enjoyment

The interviews not only revealed how participants constantly navigate, manage, and cope with unpleasant emotional reactions when challenging colour‐blindness, but also how joy and excitement expressed by the white majority play a crucial role in exercising racial power and controlling the premise of the conversation. Given participants usual commitment to anti‐racist work, this aspect became particularly evident when exploring which conversations they, to the best of their abilities, attempted to shy away from. Like Redwan, a community facilitator explains:

I usually don’t entertain them for too long. I’m usually like, “Yeah, sure. I get what you mean. Interesting. Here’s an article you can read” … Either I end the conversation pretty quickly or I just let them ramble on without engaging. I remember one time—it was actually kind of awful—when some friends and I were at a party. A girl I didn’t know that well came over; this was about a month after George Floyd had been killed. She talked to me for like 40 minutes about how horribly racist her family was and how she absolutely wanted to show solidarity with me. And I’m standing there like, ‘I’m at a party’ (laughs). She wasn’t unpleasant or anything, but if I haven’t initiated the conversation… It was just that she talked for so long, nonstop, about herself—about how great of a person she was because she had told her grandfather, ‘You know what, Black people are people too’. I was like, ‘Wow, revolutionary’ (laughs).

Redwan illustrates how it seems to be an emotional block in conversations between the racialised and non‐racialised population, also in interactions initiated by white people. As a Black person, Redwan recalls how: ‘[racism] was something we always talked about. They [parents] were like, it sucks to say this to children, but you must fight twice as hard as everyone else. You simply can't afford to give up’. This account might explain why the enthusiasm and joy expressed by the girl did not circulate and attach to him, as the lack of reflections on racial oppression until the BLM uprisings stands in stark contrast to how fear and anxiety have circulated within Redwan's family and attached to his body (Ahmed 2004). However, because this interaction unfolds in a colour‐blind context where the majority claim liberal values and, in this case, even anti‐racist sympathies, the unequal distribution of emotions remains invisible to the dominant other.

In this story, it is also worth noting how the girl does not seem to pay much attention to Redwan's experience of the interaction, as she talks ‘nonstop’ for 40 min about herself. In attempting to unpack this dynamic more closely, Hartman's (2024) notion on how whites feel entitled to enjoyment from Black people's presence proved useful. In this view, the act of initiating a conversation about racism simply for pleasure should not be regarded as innocent, but rather as an active choice of ignoring the racialised others' emotional experience while simultaneously seeking enjoyment in this group's suffering. Alex, whose work focuses on exposing illegal adoption industries, found these situations particularly challenging:

If he asks where I’m really from, I just get a bit exasperated and say that I’m from [birth country]. Then they always go, ‘Oh, okay…’ Sometimes people get a little curious—this could be at a pre‐party, you know. Then they might ask, ‘Oh, interesting. When did you come here?’ And when I answer, ‘I grew up here, I’m adopted’, they always respond with this exaggerated tone, ‘Ah, you’re adopted! Have you been back to your country?’ (sighs) I can’t be bothered with this right now. I’m sitting at a pre‐party, and it’s only nine o’clock. Am I really supposed to talk about my adoption story with someone I met five minutes ago? At that point, I completely steer the conversation away. I change the subject, but they don’t even realise I’m doing it. Inside, I’m boiling, but in the moment, I just say, ‘Yeah, I’m from [birth country], but it’s fine’.

Alex's story highlights the importance of context when studying the relationship between enjoyment and racial domination in colour‐blind societies like Norway. As their story illustrate, participants revealed a pattern in which private, sensitive, and often traumatic events were frequently brought up by the racially dominant in contexts that were initially meant to be relaxed and enjoyable. Thus, the expectations of Alex to share their adoption story 5 minutes into a conversation with a stranger indicate what I argue could be seen as an expression of how violence and enjoyment are intertwined. But because the process of exercising control over emotions, subjectivity, and stripping away humanity is expressed subtly, constantly shifting depending on the specific context, this form of dominations may appear more innocent.

These narratives are interesting, also because they contradict the emotional reactions of anger, shame, and discomfort presented in the previous section. In this important sense, participants' stories reveal that it is not the topic of racism itself that is perceived as too complex or emotionally demanding; rather, it becomes so when white people do not control the premise of the conversation. This tendency often plays out in public talks on racism as well, like Chris remarks: ‘All the white people were very happy with my lecture—that cannot be the standard for whether it was good or not. I would even say that if you spoke to an almost entirely white audience about racism and everyone was super happy afterwards, you probably didn't do it right’. Similarly, Anwar shared his thoughts on what he terms ‘fun chats’ about racism:

At work, we had a group visiting to confront their own racist attitudes. It was actually quite nice, but once again the conversation turned to something like, ‘Back in the day you could say the n‐word, but you can’t anymore’. And I was just like, ‘Seriously? […] Fortunately, it wasn’t me leading that conversation, it was my white colleague. And he ended up talking about his uncle who had some ‘special opinions’. But you see, instead of discussing how the Norwegian state funds [right‐wing extremist organisation], meaning we have state‐backed racism, you start talking about your uncle (laughs). And when that happens, I get so angry, but I don’t always have the energy to deal with being the ‘angry immigrant’. So, I just go make myself a coffee. I can’t always be him ‘Ah, there’s Anwar again. Angry and talks about racism—wow, I guess it’s Tuesday’ (laughs).

Anwar describes how subscribing to a simplistic and individualised understanding of racism while simultaneously distancing oneself from the issue, evokes interest, enthusiasm, and engagement among the visitors and his colleague. These emotions circulate easily within the institutional space and between bodies racialised as white but do not, however, ‘stick’ to him (Ahmed 2004). Anwar was not alone in withdrawing from such conversations, revealing the complex, subtle, almost impossible yet violent emotional terrain of colour‐blindness.

## 7 ‘Sharing the Discomfort’: Disrupting Notions of Racism in Public Spaces

The previous stories highlight how emotions expressed by the white majority work to exercise power in defining how and when to talk about racism. In turn, participants were often left with an overall impression of disempowerment, leading them to withdraw from conversations. Nevertheless, as actors in anti‐racist communities, they were also actively resisting such domination. They were constantly negotiating whose emotions should be considered most valid, thereby potentially altering the very foundation of the conversation.

Participants highlighted the importance of allowing oneself to feel and express emotions. As described in earlier studies of everyday‐racism (Essed 1991), participants frequently recalled how their feelings were often dismissed or minimised when sharing stories of racial abuse. Like Amira, an artist, who reflects how this affected her well into adulthood: ‘When I brought up examples of racism, I was so afraid of portraying myself as whiny—like, poor me [being a refugee], having gone through this and that’. Yet, all participants found themselves attempting to identify and challenge this economy of emotions today, albeit being in different stages of the process. Lisa, for example, explicates how internalisation of her mother's feelings still prevents her from calling out racism:

At least for me as a child, if I said something to stand up for myself, to call out racism, I ended up protecting the other person instead. I avoided saying anything… My mom, because she would get upset. I would take it inward and that made me feel bad and caused me stress. [Me: When you do that over and over again, it builds up] Exactly. Not allowing yourself to feel emotions is not good, and that’s kind of what I did in my interaction with my friend too. Instead of letting him feel sad or upset when I said, ‘Now listen to how minority stress works’.

In this quote, the narrative element of negotiating the importance of one's own emotions when ‘calling out racism’ carries significant meaning. Lisa articulates how she often regards other people's emotions as more important than her own, a tendency she understands to be the main factor preventing her from sharing with her friend how minority stress shapes her everyday life. In this sense, she narrates how the act of ‘allowing yourself to feel emotions’ is, on its own, associated with resistance. Jasmin offers a similar reflection, though more explicitly expressed:

Yes, [conversations about racism at parties] happen all the time for all of us. Then I get angry and not particularly pedagogical at all. I might snap and ridicule people and um, make it very clear that this is not an appropriate conversation to have. It is extremely disrespectful. I think it feels good to just put my foot down. But it’s also a way of showing that I have full human dignity and the right not to have that conversation at a party. Ultimately, it’s not my problem—it’s yours.

From the perspective that entitlement to enjoyment and racial dominance are intertwined (Hartman 2024), expressing anger becomes an act of refusing to entertain. Jasmin was not alone in highlighting the importance of expressing emotions to reclaim human dignity. In effect, these stories resonate with and support a process that collectively identify an economy of emotions that is not simply bound to the contemporary, but simultaneously operating as a constitutive force embedded within colonial dynamics. This reveals the importance of being part of a community that is capable of validating, insisting on, and reframing the importance of emotions, especially when otherwise neglected by society unaware of its colonial past.

When challenging colour‐blindness in public spaces, several participants highlighted the importance of withstanding discomfort. Instead of ‘carrying the discomfort alone’ as expressed by Emma, this responsibility should be equally shared by everyone involved. Paz, an artist, puts it simple: ‘It's not like we don't find this uncomfortable to talk about—we're just used to it. Maybe they need to get used to it too’. This understanding was expressed discreetly, such as not automatically taking social responsibility in uncomfortable situations, refraining from explaining ‘basic knowledge’ of racism, or not caring about presenting issues of racism in easy and enjoyable ways. Still, I would argue that these subtle, everyday practices are active ways of resisting, as they were carried out collectively with the explicit purpose of imposing discomfort on the dominant other. Taylor, for example, explains why they now find it more meaningful to confront colleagues' ignorance about racial structures at their workplace:

I think they understood it to some extent, but it's not really that important to me. What is important is to feel that I'm not walking around carrying that discomfort alone. Not accepting their premise, and they should know that I don't. Whatever arises there, I should not carry alone.

Similarly, Iris reflects on how taking part in human rights advocacy over time impacts her:

If you are going to stand up against racism, you won’t always be liked—that’s impossible. I try to do it in a charming way. When I give lectures, I use a lot of charm and charisma, joke around and such. It’s about keeping people open and receptive. The older I get, the more I also see that sometimes I have to be tougher and clearer, and I actually be comfortable with not being liked. The only thing I really need in my job is to be respected.

In slightly different ways, Taylor and Iris express how sharing the same emotional experience when discussing racism with whites is often considered more meaningful than reaching an agreement itself. This pattern reveals how participants challenge colour‐blindness in meaningful ways that may facilitate change. Through practices that acknowledge their own unpleasant and distressing emotions while simultaneously preventing the circulation of potentially positive emotions among the racially dominant group (Ahmed 2004), they are challenging the economy of emotions that cements colour‐blind language and discourses in the first place.

A noteworthy aspect in Iris' account is how the transformation of becoming ‘tougher and clearer’ is associated with years of experience. This observation was particularly evident among the most experienced anti‐racists—all describing the ability to express emotions and withstanding discomfort as a constant and evolving ‘learning process’. As Tao, a grassroot activist, reassures me by the end of the interview: ‘The more you do it, the more you realise that it’s not that dangerous. And usually, it's the ones it concerns who find it most uncomfortable’. This highlights the transformative potential of the Norwegian anti‐racist communities in disrupting colour‐blindness in public spaces. By collectively taking up emotional space and speaking truth to power that usually shuts down communication, participants are laying ground for constructive dialogues on racism across racial divides.

## 8 Concluding Discussion

Following the work of Bonilla‐Silva (2021) and his concept of colour‐blindness, I have argued that an unequal distribution of emotions across racial divides works as a central mechanism in the development of colour‐blind ideology, thus calling attention to the need to revisit the relationship between micro‐level interactions and colour‐blindness beyond the realm of language. Existing literature predominantly describes how narratives and storylines reflect and reproduce colour‐blindness, based on the argument that the language nowadays is flexible, subtle, and inconsistent enough to hide statements denying systemic racism. However, when analysing attempts to challenge such domination—a focus largely overlooked in this literature—language alone did not explain why communication across racial divides often falls short.

Based on my analysis, I argue that different emotional constellations cement the divide in understanding found on a discursive level. In colour‐blind societies where the white majority claim liberal values and in some cases even anti‐racist sympathies, an unequal distribution of emotions across racial divides nonetheless perpetuates a dynamic in which white people exercise racial power and control the premise of the conversation. These insights echo Ahmed's (2004) argument on how emotions work as an economy. From a relational perspective, emotions expressed by the white majority are extractive, cohesive, and violent, (re)enforcing racial relations and hierarchies. Hence, the only way of resisting such racially embedded dynamics is to forego the emotional work of pleasing the dominant other and challenge the power that creates the very foundation for why communication fail.

Hartman's (2024) analysis of whites' entitlement to enjoyment further illustrates how the unequal distribution of emotions ultimately stems from the colonial dichotomy between the human and the Other, locating Norway within global and interconnected processes of coloniality. This deepens our understanding of the internal dynamics underpinning the relatively stable nature of colour‐blindness (e.g., Doane 2017; Hughey et al. 2015), showing how the anti‐racists not only are temporally situated in the present, but also tethered to the colonial past when interacting with the white majority. A notable finding of this study is that the unequal distribution of emotions would appear more recognisable across racial divides had it unfolded in a social context where colonial dynamics were less concealed. This presents a temporal paradox that would not be revealed through an analytical focus on language alone, yet leaves the anti‐racists within a complex emotional terrain of confusing, subtle, yet violent social interactions that are impossible to resist unless one can manage and navigate seemingly contradictory temporal dimensions at once.

Importantly, and at the same time, the nationally grounded analysis of colour‐blindness highlights the implications of the Norwegian case for broader literature and research, especially in efforts to establish a critical global theory (see Beaman and Petts 2020; Doane 2017). A central point made by Bonilla‐Silva (2021) is that whites are nowadays linguistically in a better position to deny systemic racism based on the belief that we now have transcended the disabling racial divisions of the past. While this argument resonates with several Western countries, this current study offers an interesting contrast, as the colonial past has never been recognised as part of the Norwegian national narrative in the first place (McEachrane 2014). In following Doane's (2017) argument on how colour‐blindness is constantly developing dependent on the context, this might explain why the need to develop a sophisticated language able to deny systemic racism (so far) has been less necessary. In keeping with this logic, the anti‐racists offer fresh insights into possibilities for change, highlighting the potential in challenging the economy of emotions as a sustainable form of resistance that remains resilient to the constant and ever‐shifting flexibility of the language across Western national borders.

On this matter, it is important to recognise that the tendency of claiming colonial innocence is prevalent across all Western societies. For instance, through her concept of ‘white innocence’, Wekker (2016) argues that the Netherlands is experiencing a collective amnesia regarding its colonial past, serving to mask present‐day racism. Similarly, the UK shows a remarkable reluctance in recognising its role in race‐making processes, as well as accepting the structural inequalities that persist because of colonialism (Bhambra 2024). And even the US, often depicted as the horror example that other European countries like distance themselves from, is currently undergoing an enormous backlash in which the history of colonialism and racism is actively refused and banned in public education (Butler 2024). Combined with the findings outlined here, these patterns demonstrate the scholarly relevance of including emotions within the literature on colour‐blindness more broadly, particularly in efforts to disentangle and confront the contemporary forms of backlash that define our current historical moment.

This article shows how emotions work as a central mechanism in both (re)producing and challenging colour‐blind ideology; however, it has several limitations. Further research would enrich knowledge on the role of emotions by including people of colour *outside* anti‐racist communities, particularly among youths who likely have fewer opportunities to learn about the history of colonialism compared with those who grew up in the pre‐colour‐blind era. Additionally, because digital platforms and virtual communication connect ideas across national borders at a faster pace than ever before, analysing the digital public sphere as a circulation of emotions can illuminate whether these platforms operate as spaces that reinforce setback or enable resistance to the development of colour‐blind ideology.

Despite these limitations, this study disrupts a dominant notion, both in academia and the public, that language (or lack thereof) is the primary barrier to why reaching a shared understanding often fail. In our current political climate, this perspective is important because it offers an alternative approach to facilitating constructive dialogue on racism, if one truly wants to understand the others' worldview. I conclude by arguing that the simple act of acknowledging how people might have different emotional experiences across the racial line could, in itself, lead to a more fruitful conversation on racism. Obviously, the act of addressing emotions alone will not have any impact in eradicating structural inequalities as such; however, it may be a first step in moving toward a real dialogue on racism—insights that become increasingly important in times where different worldviews seem to be drifting further apart.

## Funding

The author has nothing to report.

## Ethics Statement

The project has obtained ethical approval by SIKT, the Norwegian Agency for Shared Services in Education and Research (approval number 833586).

## Conflicts of Interest

The author declares no conflicts of interest.

## Data Availability

The author confirms that the data supporting the findings of this study are included within the article. However, full transcripts of the interview data are not publicly available, as participants of this study did not consent to data sharing and such disclosure could compromise their privacy.
